# 

**Published:** 1857-07

**Authors:** 


					﻿No. II.l
JULY.
[Vol. XIII.
BUFFALO
MEDICAL JOURNAL
AND
Ikuictu
GT
MEDICAL AND SURGICAL SCIENCE.
SANFORD B. HUNT, M. D.,
EDITOR.
BUFFALO, N. Y.:
E . R. JEWETT & CO.’S STEAM PRESS.
Commercial Advertiser Buildings.
1857.
Price. $2.00 in advance- or $2.50 at the end of three months.
				

